# Complete genome sequence and whole-genome phylogeny of *Kosmotoga pacifica* type strain SLHLJ1^T^ from an East Pacific hydrothermal sediment

**DOI:** 10.1186/s40793-016-0214-2

**Published:** 2017-01-05

**Authors:** Lijing Jiang, Stéphane L’Haridon, Mohamed Jebbar, Hongxiu Xu, Karine Alain, Zongze Shao

**Affiliations:** 1 Key Laboratory of Marine Genetic Resources, Third Institute of Oceanography, SOA, Xiamen, 361005 China; 2 State Key Laboratory Breeding Base of Marine Genetic Resources, Xiamen, 361005 China; 3 Key Laboratory of Marine Genetic Resources of Fujian Province, Xiamen, 361005 China; 4 Université de Bretagne Occidentale, UMR 6197-Laboratoire de Microbiologie des Environnements Extrêmes (LM2E), Institut Universitaire Européen de la Mer (IUEM), rue Dumont d’Urville, 29 280 Plouzané, France; 5 CNRS, UMR 6197-Laboratoire de Microbiologie des Environnements Extrêmes (LM2E), Institut Universitaire Européen de la Mer (IUEM), rue Dumont d’Urville, 29 280 Plouzané, France; 6 Ifremer, UMR 6197-Laboratoire de Microbiologie des Environnements Extrêmes (LM2E), Technopôle Brest-Iroise, BP70, 29 280 Plouzané, France

**Keywords:** Marine, Hydrothermal vent, *Thermotogales*, Chemoorganoheterotroph, Thermophile

## Abstract

*Kosmotoga pacifica* strain SLHLJ1^T^ is a thermophilic chemoorganoheterotrophic bacterium isolated from a deep-sea hydrothermal sediment. It belongs to the physiologically homogeneous *Thermotogaceae* family. Here, we describe the phenotypic features of *K. pacifica* together with its genome sequence and annotation. The chromosome has 2,169,170 bp, organized in one contig. A total of 1897 candidate protein-encoding genes and 177 RNA genes were identified. The 16S rRNA gene sequence of this strain is distantly related to sequences of some relatives classified in the same genus (*K. olearia* 7.02% and *K. shengliensis* 7.83%), with dissimilarity percentages close to the threshold generally described for genus delineation. Nevertheless, the percentage of conserved proteins (POCP), which is much higher than 50% (around 70%), together with phenotypic features of the isolates, confirm the affiliation all *Kosmotoga* species described so far to the same genus.

**Electronic supplementary material:**

The online version of this article (doi:10.1186/s40793-016-0214-2) contains supplementary material, which is available to authorized users.

## Introduction

The phylum *Thermotogae* is currently composed of 50 species spread across 13 genera, distinguishable mainly by their characteristic outer membrane known as the ‘toga’. These genera are named *Athalassotoga* *,* *Defluviitoga* *,* *Fervidobacterium* *,* *Geotoga* *,* *Kosmotoga* *,* *Marinitoga* *,* *Mesoaciditoga* *,* *Mesotoga* *,* *Oceanotoga* *,* *Petrotoga* *,* *Pseudothermotoga* *,* *Thermosipho* and *Thermotoga* [1–12]. They are grouped into 5 families [1, 10]: (i) *Thermotogaceae*, comprising the genera *Thermotoga* and *Pseudothermotoga*; (ii) *Fervidobacteraceae*, comprising the genera *Fervidobacterium* and *Thermosipho*; (iii) *Petrotogaceae*, comprising the genera *Petrotoga* *,* *Defluviitoga* *,* *Geotoga* *,* *Marinitoga* and *Oceanotoga*; (iv) *Kosmotogaceae*, comprising the genera *Kosmotoga* and *Mesotoga*; and (v) *Mesoaciditogaceae*, comprising the genera *Mesoaciditoga* and *Athalassotoga*. The first representatives of this phylum described from the mid-1990s were all neutrophilic, thermophilic or hyperthermophilic fermentative bacteria from a range of hot anaerobic microbial environments such as deep-sea and terrestrial vents, anaerobic digesters or oil reservoirs. They are relatively homogeneous in terms of physiology. In the last few years, the description of the genera *Mesotoga* *,* *Mesoaciditoga* and *Athalassotoga*, corresponding to three divergent lineages among the *Thermotogae*, showed that there are also representatives of this order that grow under mesophilic or slightly acidic conditions [1, 7, 8]. The different genera of *Thermotogae* display different tolerances to oxygen and salts, and can produce L-alanine or reduce different sulfur species to prevent the toxic effect of H_2_ produced during fermentation. Phylogenetic analyses of the 16S rRNA gene and of concatenated ribosomal proteins place *Thermotogae* as a sister group to *Aquificales*, representing a deeply-branching lineage of the bacterial tree that emerges close to the first delineation between bacterial and archaeal branches [13]. However, the evolutionary history of these bacteria is also characterized by numerous lateral gene transfer events with *Firmicutes* and with *Thermococcales* [13, 14].

The genus *Kosmotoga* was proposed by DiPippo et al. in 2009 [5] and belongs to the family *Kosmotogaceae* *,* one of the five families of the phylum *Thermotogae*. The genus is currently composed of four type species, *K. olearia* [5], *K. arenicorallina* [15], *K. shengliensis* [15] and *K. pacifica* [16]. *Kosmotoga* species have been isolated from oil reservoirs as well as shallow and deep-sea hydrothermal vents. Strain SLHLJ1^T^ (=DSM 26965^T^ = JCM 19180^T^ = UBOCC 3254^T^ =MCCC 1A00641^T^) is the type strain of the species *K. pacifica*, which was isolated from sediments of an active hydrothermal vent on the East Pacific Rise (102°55′W, 3°58′S) [16]. Here, we present a summary of the physiological features of *K. pacifica* SLHLJ1^T^, together with a description of the complete genomic sequence and annotation. A brief genomic comparison was made between *K. pacifica* SLHLJ1^T^ and *K. olearia* TBF 19.5.1^T^ and we also calculated (i) ANI and (ii) POCP values among pairs of genomes of *Thermotogae* for which complete genomic sequences were available.

## Organism information

### Classification and features

**Fig. 1:**
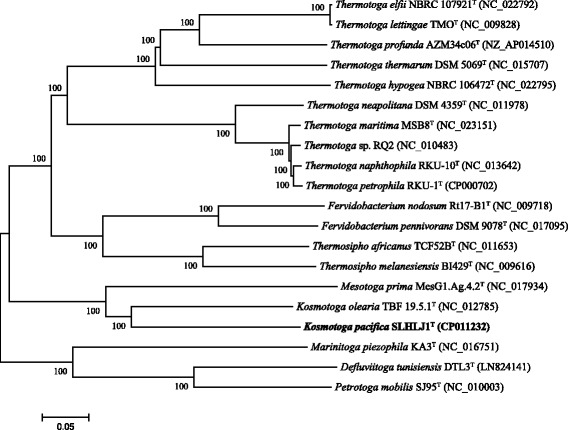
Phylogenetic tree indicating the position of *K. pacifica* strain SLHLJ1^T^ relative to other type and non-type strains with complete genome sequences within the phylum *Thermotogae*. The tree was constructed by the neighbor-joining method using 499 core genes (approximately 163,000 amino acid sequences). Bootstrap values (in %) are based on 500 replicates and are shown at the nodes with >50% bootstrap support. The scale bar represents 5% sequence divergence

Strain SLHLJ1^T^ was isolated by repeated streaking on plates as described elsewhere [16]. In this study, a whole-genome phylogeny of the *Thermotogae* lineage was constructed based on the core genome (499 core genes) from 20 complete genomes. The core genes were chosen based on identified orthologous genes, which were also single-copy genes from 20 genomes (Additional file 1: Table S1). The result indicated that *K. pacifica* SLHLJ1^T^ was affiliated to the genus *Kosmotoga*, which formed a deep branch in the phylogenetic tree constructed with the neighbor-joining algorithm (Fig. 1). *K. pacifica* SLHLJ1^T^ was closely related to *K. arenicorallina*, sharing 97.93% 16S rRNA gene sequence similarity, and was distantly related (<93%) to the other species of the genus *Kosmotoga*. Phylogenetic comparison of 16S rRNA gene sequences of *K. pacifica* SLHLJ1^T^ and other *Thermotogae* also supported the result that *K. pacifica* SLHLJ1^T^ clusters with other *Kosmotoga* species (Additional file 2: Figure S1) [16].

**Fig. 2:**
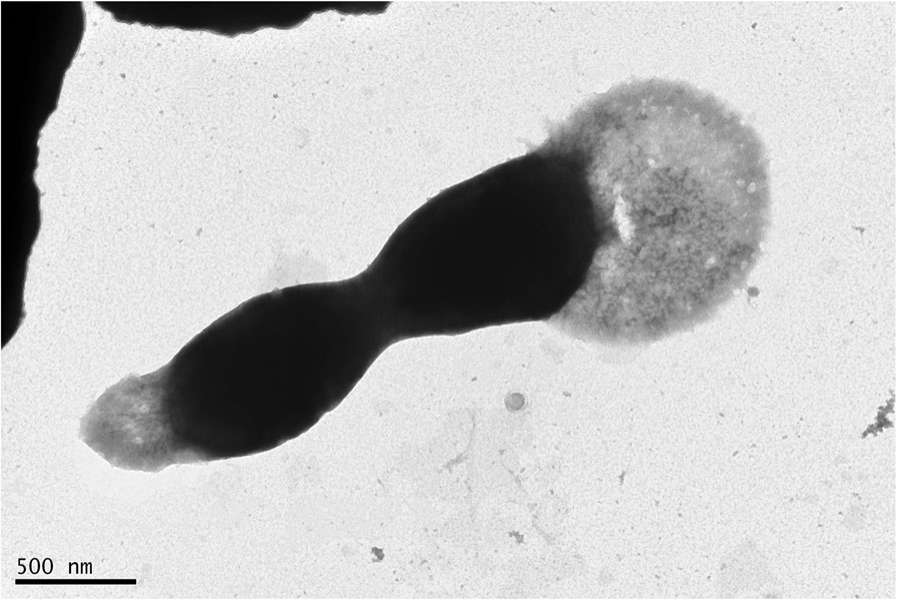
Transmission electron micrograph of *K. pacifica* strain SLHLJ1^T^, showing the toga

*K. pacifica* SLHLJ1^T^ cells are Gram-negative non-motile short rods or ovoid cocci (~1 μm long by ~0.6 μm wide) surrounded by a typical toga. They appear singly or occasionally in chains of 3–4 cells within the sheath (Fig. 2). Spores were never observed. Strain SLHLJ1^T^ grows between 33 and 78 °C, but the optimal growth temperature is 70 °C. Growth occurs under strictly anaerobic and obligate chemoorganoheterotrophic conditions. A small amount of yeast extract is required for growth. The following substrates support growth in the presence of 0.02% yeast extract: peptone, brain–heart infusion, tryptone, glycerol, maltose, xylose, glucose, fructose, cellobiose, trehalose, lactate, propionate and glutamate. The strain can reduce L-cystine and elemental sulfur [16]. A summary of the classification and general features of *K. pacifica* SLHLJ1^T^ is presented in Table 1.

**Table 1:** Classification and general features of *K. pacifica* SLHLJ1^T^ [12]

MIGS ID	Property	Term	Evidence code^a^
		Domain *Bacteria*	TAS [17]
		Phylum *Thermotogae*	TAS [18, 19]
		Class *Thermotogae*	TAS [18, 20]
	Current classification	Order *Kosmotogales*	TAS [18, 21]
		Family *Kosmotogaceae*	TAS [18, 22]
		Genus *Kosmotoga*	TAS [12, 23]
		Species *Kosmotoga pacifica*	TAS [16]
		Type strain SLHLJ1^T^	TAS [16]
	Gram stain	Negative	TAS [16]
	Cell shape	Coccobacilli with a ‘toga’ (a sheath-like structure)	TAS [16]
	Motility	non-motile	TAS [16]
	Sporulation	Non-sporulating	TAS [16]
	Temperature range	33-78 °C	TAS [16]
	Optimum temperature	70 °C	TAS [16]
	pH range; Optimum	5.5-8.5;7	
	Carbon source	Yeast extract, peptone, brain–heart infusion, tryptone, glycerol, maltose, xylose, glucose, fructose, cellobiose, trehalose, lactate, propionate and glutamate	TAS [16]
	Energy metabolism	Chemoorganoheterotrophic	TAS [16]
MIGS-6	Habitat	Hydrothermal vent environment	TAS [16]
MIGS-6.3	Salinity	0.5-6% NaCl (w/v)	TAS [16]
MIGS-22	Oxygen requirement	Anaerobic	TAS [16]
MIGS-15	Biotic relationship	Free living	TAS [16]
MIGS-14	Pathogenicity	None	NAS
MIGS-23.1	Isolation	Sediment	TAS [16]
MIGS-4	Geographic location	East Pacific Rise	TAS [16]
MIGS-5	Sample collection time	July 2011	TAS [16]
MIGS-4.1	Latitude	3°58′S	TAS [16]
MIGS-4.2	Longitude	102^o^55′W	TAS [16]
MIGS-4.3	Altitude	-2891 m	TAS [16]

^a^ Evidence codes - TAS: Traceable Author Statement (i.e., a direct report exists in the literature); NAS: Non-traceable Author Statement (i.e., not directly observed for the living, isolated sample, but based on a generally accepted property for the species, or on anecdotal evidence). These evidence codes are from the Gene Ontology project [24]. ^*^ The rank of phylum is not covered by the Rules of the International Code of Nomenclature of Prokaryotes

## Genome sequencing information

### Genome project history

This organism was selected for sequencing based on its phylogenetic position. The complete genome sequence was deposited in GenBank under the accession number CP011232. Sequencing, finishing and annotation of the *K. pacifica* SLHLJ1^T^ genome were performed by the Shanghai Majorbio Bio-pharm Technology Co., Ltd (Shanghai, China). Table 2 presents the main project information and its association with MIGS version 2.0 compliance [25].

**Table 2:** Project information

MIGS ID	Property	Term
MIGS-31	Finishing quality	Finished
MIGS-28	Libraries used	Two genomic libraries: one 454 PE library (3 kb insert size) and one Illumina PE library (500 bp insert size)
MIGS-29	Sequencing platforms	Illumina Miseq, 454 GS FLX Titanium
MIGS-31.2	Fold coverage	564 × Illumina; 112 × 454 3 K-PE
MIGS-30	Assemblers	Newbler version 2.8
MIGS-32	Gene calling method	NCBI PGAP pipeline
	Locus Tag	IX53
	Genbank ID	CP011232.1
	GenBank Date of Release	June 3, 2015
	GOLD Project ID	Gp0119521
	BIOPROJECT	PRJNA256122
MIGS-13	Source material identifier	UBOCC 3254 and MCCC 1A00641
	Project relevance	Thermophile, GEBA

### Growth conditions and DNA isolation

Strain SLHLJ1^T^ was grown anaerobically for 24 h at 70 °C in 50 mL DSMZ medium 282 (with yeast extract as a carbon and energy source), supplemented with 12 g/L L-cystine. DNA was isolated from the liquid phase without L-cystine, using a standard phenol/chloroform/isoamyl alcohol extraction protocol [26]. The quality and quantity of the extracted DNA were analyzed using agarose gel electrophoresis and NanoDrop. A total of around 20 μg DNA was obtained.

### Genome sequencing and assembly

The genome was sequenced using a combination of an Illumina MiSeq (2 × 300 bp) and 454 sequencing platforms. Libraries were prepared in accordance with manufacturer’s instructions. The Newbler V2.8 software package was used for sequence assembly and quality assessment [27]. The draft genome sequence was generated using 454 data. The 454 draft assembly was based on 243,758,031 bp 454 draft data. Newbler parameters were -consed, -a 50, -l 350, -g, -m, and -ml 20. The Phred/Phrap/Consed software package [28] was used for sequence assembly and quality assessment in the subsequent finishing process. Illumina reads were used for gap-filling, correcting potential base errors and increasing consensus quality. Gaps were then filled in by sequencing the PCR products using an ABI 3730xl capillary sequencer. A total of four additional reactions were necessary to close gaps and to improve the quality of the finished sequence. Together, the combination of the Illumina and 454 sequencing platforms provided 676 × coverage of the genome. The final assembly contained 637,426 pyrosequences and 4,870,336 Illumina reads.

### Genome annotation

The protein-coding genes, structural RNAs (5S, 16S, and 23S), tRNAs and small non-coding RNAs were predicted using the NCBI PGAP server online [29]. The functional annotation of predicted ORFs was performed using RPS-BLAST [30] against the COG database [31] and Pfam database [32]. The TMHMM program was used for gene prediction with transmembrane helices [33] and the signalP program for gene prediction from peptide signals [34]. ANI values were calculated using JSpecies software [35] and the ANI tool of the Integrated Microbial Genome (IMG) system [36]. POCP indexes were calculated as described elsewhere [37].

## Genome properties

**Fig. 3:**
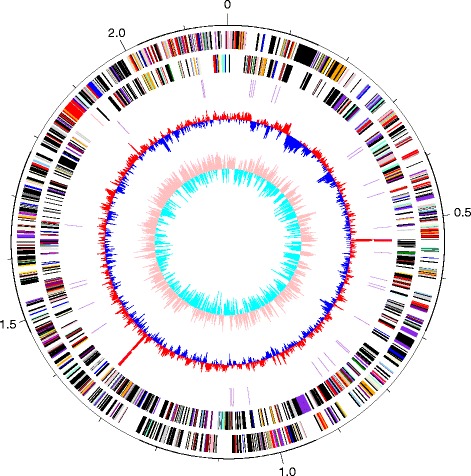
Graphical map of the chromosome of *K. pacifica* strain SLHLJ1^T^. From the edge to the center: Genes on forward strand (colored by COG categories), Genes on reverse strand (colored by COG categories), RNA genes (tRNAs purple and rRNAs red), GC content and GC skew

The properties and statistics about the genome are summarized in Table 3. The genome is organized in one circular chromosome of 2,169,170 bp (42.52% GC content). In total, 2074 genes were predicted, 1897 of which were protein-coding genes, and 177 of which were RNA genes; 124 pseudogenes were also identified. Most protein-coding genes (83.75%) were assigned putative functions and the remaining ones were annotated as hypothetical proteins. The distribution of genes between COG functional categories is presented in Table 4 and Fig. 3.

**Table 3:** Genome statistics

Attribute	Value	% of Total
Genome size (bp)	2,169,170	100.0
DNA coding (bp)	1,814,445	83.65
DNA G + C (bp)	922,242	42.52
DNA scaffolds	1	
Total genes	2074	100.00
Protein coding genes	1897	91.47
RNA genes	177	8.53
Pseudo genes	124	5.98
Genes in internal clusters	ND	ND
Genes with function prediction	1737	83.75
Genes assigned to COGs	1124	54.19
Genes assigned Pfam domains	1770	85.34
Genes with signal peptides	37	1.78
Genes with transmembrane helices	538	25.94
CRISPR repeats	0	0

*ND* not determined

**Table 4:** Number of genes associated with the general COG functional categories

Code	Value	% ^a^	Description
J	129	6.80	Translation, ribosomal structure and biogenesis
A	0	0.00	RNA processing and modification
K	53	2.79	Transcription
L	53	2.79	Replication, recombination and repair
B	2	0.11	Chromatin structure and dynamics
D	13	0.69	Cell cycle control, cell division, chromosome partitioning
Y	0	0.00	Nuclear structure
V	20	1.05	Defense mechanisms
T	24	1.27	Signal transduction mechanisms
M	45	2.37	Cell wall/membrane biogenesis
N	2	0.11	Cell motility
Z	0	0.00	Cytoskeleton
W	0	0.00	Extracellular structures
U	17	0.90	Intracellular trafficking and secretion, and vesicular transport
O	49	2.58	Posttranslational modification, protein turnover, chaperones
C	109	5.75	Energy production and conversion
G	128	6.75	Carbohydrate transport and metabolism
E	105	5.54	Amino acid transport and metabolism
F	40	2.11	Nucleotide transport and metabolism
H	34	1.79	Coenzyme transport and metabolism
I	26	1.37	Lipid transport and metabolism
P	80	4.22	Inorganic ion transport and metabolism
Q	14	0.74	Secondary metabolites biosynthesis, transport and catabolism
R	96	5.06	General function prediction only
S	85	4.48	Function unknown
-	773	40.75	Not in COGs

^a^ The total is based on the total number of protein coding genes in the genome

## Insights from the genome sequence

In the genome sequence of *K. pacifica* SLHLJ1^T^, a relatively large number of genes were observed to be assigned to the COG functional categories for transport and metabolism of carbohydrates (6.75%), amino acids (5.54%), translation, ribosomal structure and biogenesis (6.8%), and energy production and conversion (5.75%). Further genome analysis of *K. pacifica* SLHLJ1^T^ revealed it contained genes for the Embden-Meyerhof-Parnas pathway to convert glucose into pyruvate, but not for the complete pentose phosphate pathway and Entner-Doudoroff pathway due to the lack of several key genes (such as glucose 6-phosphate dehydrogenase and 2-keto-3-deoxy-6-phospho-gluconate aldolase). In addition, the tricarboxylic acid cycle was also found to be incomplete in *K. pacifica* SLHLJ1^T^. The strain is capable of breaking down substrates such as xylose, cellobiose or trehalose, which is not surprising since an abundance of genes coding for carbohydrate breakdown has been predicted in its genome.

Prior to this study, the only available genome for the genus *Kosmotoga* was *K. olearia* TBF 19.5.1^T^. Here, we compared the genome of *K. pacifica* SLHLJ1^T^ with *K. olearia* TBF 19.5.1^T^ (Table 5). *K. olearia* and *K. pacifica* share share 92.98% 16S rRNA gene sequence similarity based on full 16S rRNA sequences. The genome size of strain SLHLJ1^T^ is slightly smaller than that of strain TBF 19.5.1^T^. These two strains have nearly identical G + C contents: 42.52% for strain SLHLJ1^T^ against 41.5% for strain TBF 19.5.1^T^. Strain SLHLJ1^T^ has a slightly smaller gene content than strain TBF 19.5.1^T^ (2074 *vs* 2194). *K. pacifica* SLHLJ1^T^ shares 1524 orthologous genes with *K. olearia* TBF 19.5.1^T^.

**Table 5:** Comparative genomic characteristics of *K.pacifica* SLHLJ1^T^ and *K. olearia* TBF 19.5.1^T^

Genome Name	Genome size (bp)	%GC	Gene count	Protein coding	Plasmid number	rRNA	tRNAs	Orthologous genes
*K. pacifica* SLHLJ1^T^	2,169,170	42.5	2074	1897	0	6	46	1534
*K. olearia* TBF 19.5.1^T^	2,302,126	41.5	2194	2116	0	6	46	1579

**Fig. 4:**
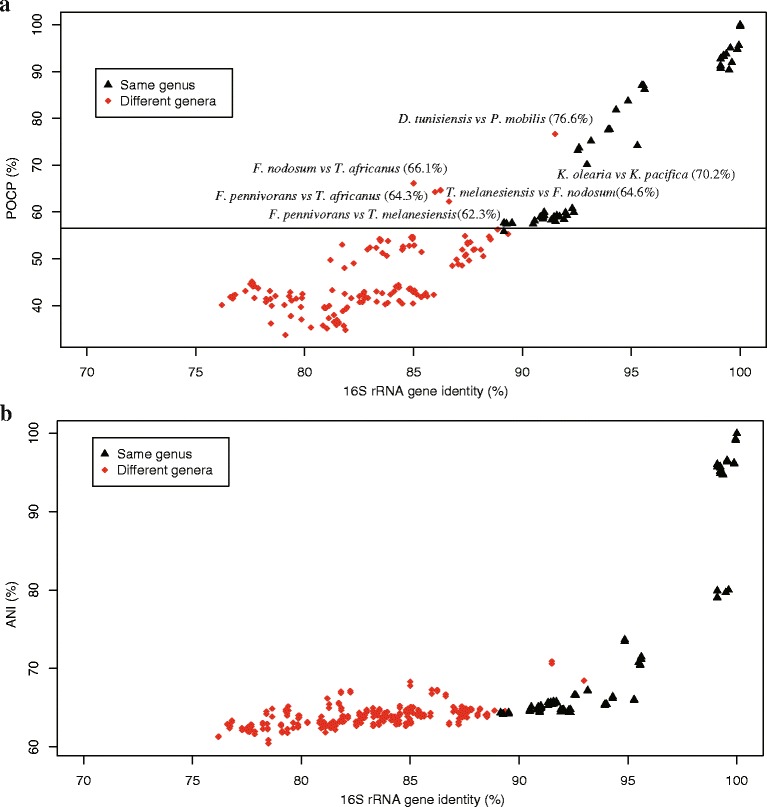
Relationships between POCP (**a**)/ANI (**b**) and 16S rRNA gene identity for pairs of genomes from different genera and the same genus within *Thermotogae*. ANI values were calculated using JSpecies software

Furthermore, we wanted to confirm the affiliation of *K. pacifica* SLHLJ1^T^ to the genus *Kosmotoga* with genomic data. Indeed, there are two lineages within the *Kosmotoga* genus (*K. pacifica* SLHLJ1^T^ and *K. arenicorallina* S304^T^ on the one hand, and *K. shengliensis* 2SM-2^T^ and *K. olearia* TBF 19.5.1 T on the other) and these are distantly related based on 16S rRNA gene sequence comparisons (they share between 91.7 and 92.4% 16S rRNA gene sequence similarity) [38]. ANI is a useful index for species circumscription [35], and it was recently proposed that a prokaryotic genus could be defined as a group of species with all pairwise POCP values higher than 50% [37]. We therefore performed these two types of analyses to address the issue of the limits of the genus *Kosmotoga*. The POCP index and ANI value between *K. pacifica* SLHLJ1^T^ and *K. olearia* TBF 19.5.1^T^ were respectively 70.2% and 68.5% (with JSpecies) (Fig. 4), or 72.5% (with the IMG system), supporting the assignment of these two isolates to two different species of the same genus.

A total of 20 complete genomic sequences belonging to the phylum *Thermotogae* are publicly available in the NCBI database, including representatives of the genera *Defluviitoga*, *Fervidobacterium*, *Kosmotoga*, *Marinitoga*, *Mesotoga*, *Petrotoga*, *Thermosipho* and *Thermotoga*. To gain a thorough understanding of the evolutionary relationships and phenotypic distances among the different groups in the *Thermotogae*, a phylogenomic analysis was conducted based on core gene sequences from these 19 genomic sequences and the one of *K. pacifica*. In addition, POCP and ANI values between pairs of strains were also calculated. Results are shown in Figs. 1 and 4. The interspecies ANI values calculated using JSpecies and IMG system software ranged from 64 to 99% and from 6 to 99%, respectively, while the intergenera ANI values were in the ranges of 60–70% and 65–86%. Thirty six percent of the intergenera ANI values overlapped with the interspecies ANI values; a result showing, in agreement with [37],that ANI cannot be used as a boundary for genus delineation. Interspecies POCP values were between 55.8 and 95.6%, with a large majority above 57%. Intergenera POCP values ranged from 33.7 to 76.6%, with a majority below 57% (Fig. 4, Additional file 3: Table S3). POCP analyses revealed that there were several high percentages of conserved proteins between representatives of different genera, such as *Defluviitoga tunisiensis* *vs* *Petrotoga mobilis* (76.6%), *Fervidobacterium nodosum* *vs* *Thermosipho africanus* (66.1%), *Fervidobacterium pennivorans* *vs* *Thermosipho africanus* (64.3%) or *Thermosipho melanesiensis* *vs* *Fervidobacterium nodosum* (64.6%). This result was surprising for us, knowing that 16S rRNA gene sequence dissimilarities among *Thermotogae* genera (>11%) are much higher than in the vast majority of bacterial orders, but that physiology is homogeneous among the *Thermotogae*, with only a few or minor differences between genera (Additional file 4: Table S2).

Representatives of the four following groups: *Defluviitoga*/*Petrotoga*, *Fervidobacterium*/*Thermotoga*/*Thermosipho*, *Marinitoga*/*Petrotoga*, and *Kosmotoga*/*Mesotoga* (two genera characterized by their distinctly different temperature ranges for growth), shared all pairwise POCP values higher than 50%, which is the pairwise POCP value suggested as a threshold for genus delineation [37]. These clusters of genera are in agreement with the well-resolved clades identified in a previous comparative genomic analysis and supported by multiple conserved signature indels [10]. The compilation of physiological and genotypic features of the different genera (Additional file 4: Table S2), together with the POCP index (Fig. 4 and Additional file 3: Table S3) and 16S rRNA phylogenetic distance (Additional file 2: Figure S1) tend to indicate that the pairs of *Defluviitoga*-*Petrotoga* and *Fervidobacterium*-*Thermosipho* representatives are less genotypically distant and also have less differentiating characteristics than the other pairs of genera. The results of POCP values together with the physiology of these taxa call into question the classification of the *Thermotogae* at the genus phylogenetic level and suggest that either (i) there might be fewer genera of *Thermotogae* than currently described, and that *Thermotogae* could be reclassified at the genus level by taking into account genomic information, evolutionary history and discriminative physiological characteristics; or (ii) the POCP might not be a sufficiently resolved genomic index for the delineation of genera within a homogeneous phenotype. In the light of these observations, it could be interesting to perform deep phylogenetic analyses of the *Thermotogae* (with a maximum of genomes) to study the evolutionary history and parallel evolution of genotypes and phenotypes within this *family*.

## Conclusions

Strain SLHLJ1^T^ is the first strain of the genus *Kosmotoga* to be isolated from the deep-sea hydrothermal vent environment. Its physiology and genetic content were compared to those of other *Thermotogae*. This comprehensive analysis showed that genomic information is necessary to understand the evolutionary relationships of the different groups in this well-defined lineage characterized by homogeneous physiology.

## Additional files

Additional file 1: Table S1. List of the core genes chosen for the whole genome phylogenetic analysis. This list is composed of 499 orthologous genes from 20 genomes within the phylum *Thermotogae*. (XLS 210 kb)
Additional file 2: Figure S1. Phylogenetic tree based on 16S rRNA gene sequences showing the position of *K. pacifica* strain SLHLJ1^T^ within the phylum *Thermotogae*. The alignment was performed with 16S rDNA sequences of related species and environmental sequences. The topology shown was obtained with the neighbor-joining algorithm. Bootstrap values (from 1000 replicates) are indicated at the branch nodes. The scale bar represents 2% sequence divergence. (PDF 462 kb)
Additional file 3: Table S3. Comparison of POCP value and 16S rRNA gene identity for pairs of genomes from different genera of *Thermotogae*. (DOCX 21 kb)
Additional file 4: Table S2. Differential characteristics of eight genera of *Thermotogae,* with genome sequences*.* Data were taken from *Defluviitoga* [2], *Fervidobacterium* [3, 39], *Kosmotoga* [5, 15, 16], *Marinitoga* [6, 40], *Mesotoga* [8], *Petrotoga* [4, 41], *Thermosipho* [10, 42], and *Thermotoga* [12, 43]. ND, No data available; +, positive; -, negative; ±, positive for some species, but not all. (DOCX 18 kb)

## Abbreviations

ANI: Average nucleotide identity
PGAP: Prokaryotic genome annotation pipeline
POCP: Percentage of conserved proteins
