# Supplementary figures and images for: Complete genome sequence and whole-genome phylogeny of Kosmotoga pacifica type strain SLHLJ1T from an East Pacific hydrothermal sediment
Source: Stand Genomic Sci. 2017 Jan 5;12:3. doi: 10.1186/s40793-016-0214-2 (PMC5217533; doi:10.1186/s40793-016-0214-2)

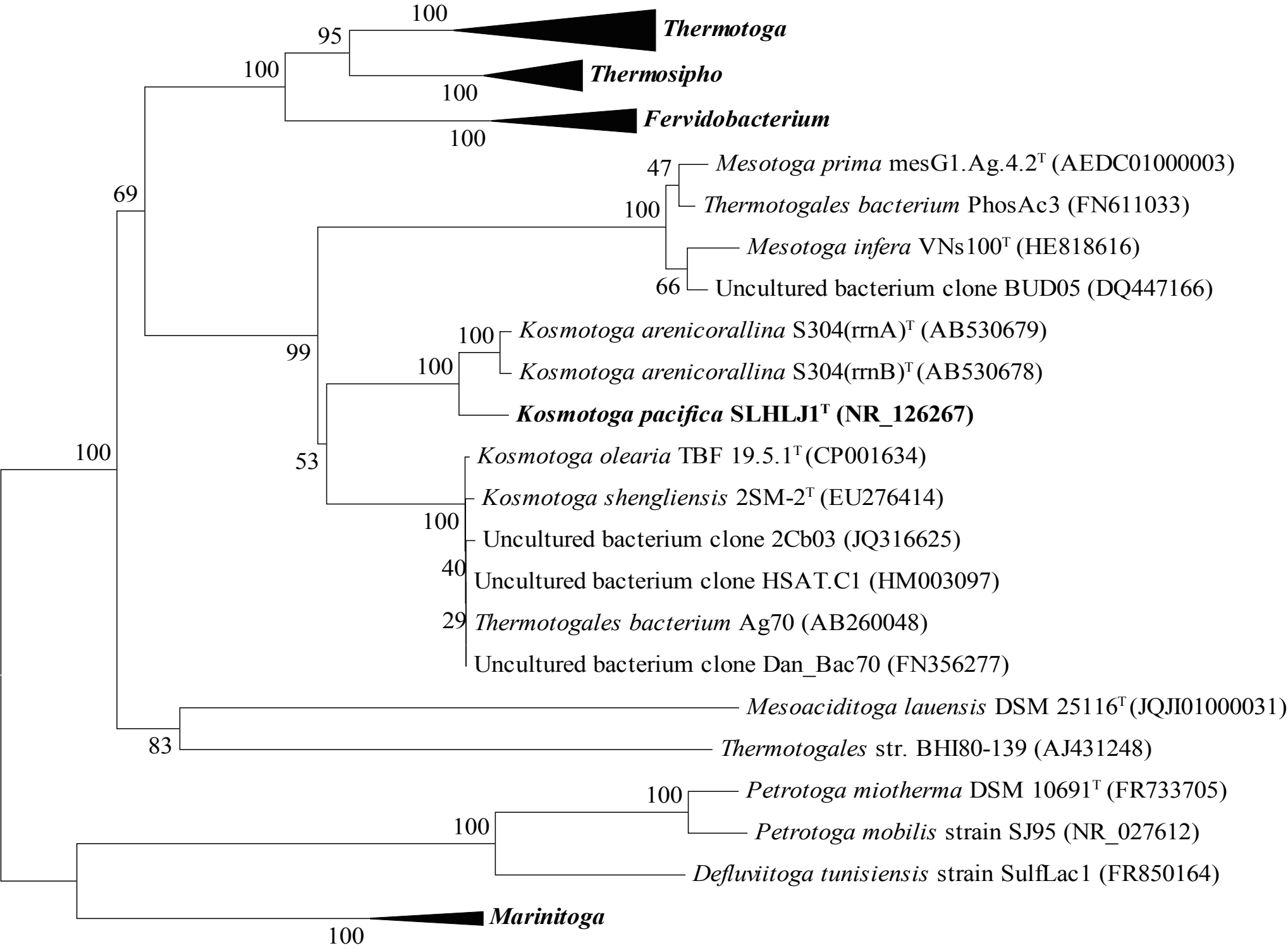

0.02

Supplement: Additional file 2: Figure S1. — Phylogenetic tree based on 16S rRNA gene sequences showing the position of K. pacifica strain SLHLJ1T within the phylum Thermotogae. The alignment was performed with 16S rDNA sequences of related species and environmental sequences. The topology shown was obtained with the neighbor-joining algorithm. Bootstrap values (from 1000 replicates) are indicated at the branch nodes. The scale bar represents 2% sequence divergence. (PDF 462 kb) [file 40793_2016_214_MOESM2_ESM.pdf]
