# Supplementary material for: Complete genome sequence and whole-genome phylogeny of Kosmotoga pacifica type strain SLHLJ1T from an East Pacific hydrothermal sediment
Source: Stand Genomic Sci. 2017 Jan 5;12:3. doi: 10.1186/s40793-016-0214-2 (PMC5217533; doi:10.1186/s40793-016-0214-2)
Supplement: Additional file 3: Table S3. — Comparison of POCP value and 16S rRNA gene identity for pairs of genomes from different genera of Thermotogae. (DOCX 21 kb) [file 40793_2016_214_MOESM3_ESM.docx]

| **Comparative Genus** | **POCP value**  **(**%**)** | **16S rRNA gene sequence identity**  **(%)** |
| --- | --- | --- |
|  |  |  |
| *Defluviitoga vs Fervidobacterium* | 41.5-41.6 | 79.6-79.9 |
| *Defluviitoga vs Kosmotoga* | 37.0-37.8 | 79.4-79.9 |
| *Defluviitoga vs Marinitoga* | 49.7 | 81.19 |
| *Defluviitoga vs Mesotoga* | 33.7 | 79.1 |
| *Defluviitoga vs Petrotoga* | 76.6 | 91.5 |
| *Defluviitoga vs Thermosipho* | 41.1-42 | 78.7-79.4 |
| *Defluviitoga vs Thermotoga* | 39.9-44.8 | 77.5-78.5 |
| *Fervidobacterium vs Kosmotoga* | 40.6-41.6 | 82.1-83.8 |
| *Fervidobacterium vs Marinitoga* | 42.5-43.3 | 81.3-81.8 |
| *Fervidobacterium vs Mesotoga* | 35.3-35.7 | 80.3-80.8 |
| *Fervidobacterium vs Petrotoga* | 42.5-42.9 | 79.3-79.9 |
| *Fervidobacterium vs Thermosipho* | 62.3-66.1 | 85-86.6 |
| *Fervidobacterium vs Thermotoga* | 50.6-54.7 | 82.9-85.4 |
| *Kosmotoga vs Marinitoga* | 40.8-41.5 | 82.7-82.8 |
| *Kosmotoga vs Mesotoga* | 55.3-56.3 | 89.4-88.9 |
| *Kosmotoga vs Petrotoga* | 39.7-40.1 | 79.1-79.8 |
| *Kosmotoga vs Thermosipho* | 42.3-43.3 | 83.9-85.9 |
| *Kosmotoga vs Thermotoga* | 40.2-44.4 | 83.3-85.6 |
| *Marinitoga vs Mesotoga* | 35.1 | 81.0 |
| *Marinitoga vs Petrotoga* | 53.0 | 81.7 |
| *Marinitoga vs Thermosipho* | 48-49 | 81.8-82.3 |
| *Marinitoga vs Thermotoga* | 37.3-42.9 | 80.9-82.7 |
| *Mesotoga vs Petrotoga* | 36.1 | 78.5 |
| *Mesotoga vs Thermosipho* | 34.8-35.7 | 81.8-81.9 |
| *Mesotoga vs Thermotoga* | 35.8-39.9 | 80.9-81.6 |
| *Petrotoga vs Thermosipho* | 42-43.1 | 78.4-79.3 |
| *Petrotoga vs Thermotoga* | 40.1-45.1 | 76.2-77.6 |
| *Thermosipho vs Thermotoga* | 48.5-54.8 | 86.8-88.5 |

**Table S3**
