# Supplementary material for: Complete genome sequence and whole-genome phylogeny of Kosmotoga pacifica type strain SLHLJ1T from an East Pacific hydrothermal sediment
Source: Stand Genomic Sci. 2017 Jan 5;12:3. doi: 10.1186/s40793-016-0214-2 (PMC5217533; doi:10.1186/s40793-016-0214-2)
Supplement: Additional file 4: Table S2. — Differential characteristics of eight genera of Thermotogae, with genome sequences. Data were taken from Defluviitoga [2], Fervidobacterium [3, 39], Kosmotoga [5, 15, 16], Marinitoga [6, 40], Mesotoga [8], Petrotoga [4, 41], Thermosipho [10, 42], and Thermotoga [12, 43]. ND, No data available; +, positive; -, negative; ±, positive for some species, but not all. (DOCX 18 kb) [file 40793_2016_214_MOESM4_ESM.docx]

**Table S2.**

| Characteristic | *Defluviitoga* | *Fervidobacterium* | *Kosmotoga* | *Marinitoga* | *Mesotoga* | *Petrotoga* | *Thermosipho* | *Thermotoga* |
| --- | --- | --- | --- | --- | --- | --- | --- | --- |
| Isolation source (s) | Sludge reactor | Terrestrial hot springs | Hydrothermal fields and oil reservoirs | Hydrothermal vents and thermal springs | Deep aquifers and harbour sediments | Oil reservoirs | Hydrothermal vents and oil reservoirs | Hydrothermal vents, oil reservoirs and bioreactors |
| Temperature Optimum (℃) | 55 | 65-80 | 60-70 | 55-65 | 37-45 | 55-60 | 65-75 | 60-80 |
| Growth at low temperatures | No growth below 37 ℃ | No growth  below 40-45℃ | No growth  below 20℃ | No growth below 25℃ | No growth below 20℃ | No growth below 30℃ | No growth below 35℃ | No growth below 45℃ |
| pH (Optimum) | 6.9 | 6.5-7.8 | 6.8-7.1 | 5.5-7.0 | 7.3-7.5 | 6.5-8.0 | 6.0-7.5 | 6.5-7.5 |
| NaCl (%) (Optimum) | 0.5 | 0-0.4 | 1.5-3.0 | 2.0-4.0 | 0.2-4.0 | 0.5-6.0 | 2.0-3.0 | 0-2.7 |
| Oxygen tolerance | *<*0.5% O2 | Strict anaerobes | Strict anaerobes  (only one species growing at 15% O_2)_ | < 4% O_2_ | < 0.5 % O_2_ | < 0.2-1% O_2_ | < 0.2-8% O_2_ | Strict anaerobes |
| Electron acceptors |  |  |  |  |  |  |  |  |
| Elemental sulfur | + | + | ± | + | + | + | ± | ± |
| Thiosulfate | + | ± | ± | ± | ± | ± | ± | + |
| Cystine | ND | ND | ± | ± | - | - | ± | ± |
| Sulfite | - | - | - | - | ± | ± | - | - |
| Sulfate | - | - | ± | - | - | - | - | - |
| DNA G+C content  (mol %) | 33.6 | 31-41 | 40.8-43.7 | 26-29 | 45.3-47.5 | 32.4-36.1 | 27-33 | 38.7-51.3 |
